# The need for introducing medication vials tailored for pediatric use. Where do we stand?

**DOI:** 10.4103/0253-7613.68441

**Published:** 2010-08

**Authors:** T. Arun Babu, C. Venkatesh

**Affiliations:** Department of Pediatrics, Sri Lakshmi Narayana Institute of Medical Sciences, Pondicherry, India

Sir,

Intravenous drug therapy is the corner stone of patient management in today’s hospital practice and the discipline of pediatrics is no exception. The drug dosage in pediatrics is usually calculated according to the body weight or surface area leading to less cumulative dose when compared to adults. Unfortunately there are no separate pediatric drug dose formulations for most of the commonly used drugs.[1] It is a common practice to use a fraction of the adult dose vials (which is often an approximate dosage) and to store the remaining drug for future use. This leads to either wastage of precious medicines or a tendency to use pre-diluted, stored and contaminated medicine which may result in adverse reactions, loss of potency and efficacy. This may also be an underlying cause for nosocomial sepsis.[2]

Intravenous drug dosage for pediatric use is often limited by the strength of the drugs available commercially, especially in developing countries.[3] Most drug companies are hesitant to manufacture pediatric drug dosage vials due to greater intricacy and cost involved in drug development, relatively small trading volume and the presence of numerous unapproved drug formulations in use. The absence of pediatric specific formulations leaves more than a third of the world’s population at an increased risk for avoidable adverse events, inappropriate dosing, non-adherence, and lack of access to novel drugs.[4]

Since in developing countries like India where sepsis is the leading cause of mortality necessitating the use of antibiotics, it is high time the medical fraternity realizes this potential lacuna in treating pediatric patients. We feel that the pharmaceutical majors should realize the urgent need to introduce commonly used drugs, especially life-saving medicines in doses suitable for pediatric use so that the basic right of every child to receive safe medication is not jeopardized.
